# Analysis of Four Aberrometers for Evaluating Lower and Higher Order Aberrations

**DOI:** 10.1371/journal.pone.0054990

**Published:** 2013-01-22

**Authors:** Fabiano Cade, Andrea Cruzat, Eleftherios I. Paschalis, Lilian Espírito Santo, Roberto Pineda

**Affiliations:** Massachusetts Eye and Ear Infirmary, Harvard Medical School, Boston, Massachusetts, United States of America; Massachusetts Eye & Ear Infirmary, Harvard Medical School, United States of America

## Abstract

**Purpose:**

To compare the measurements of lower and higher order aberrations (HOA) of 4 commonly used aberrometers.

**Setting:**

Massachusetts Eye & Ear Infirmary, Boston, USA.

**Design:**

Prospective, cross-sectional study, in a controlled, single-blinded fashion.

**Methods:**

Multiple readings were obtained in 42 eyes of 21 healthy volunteers, at a single visit, with each of the following aberrometers: Alcon LADARWave®, Visx WaveScan®, B & L Zywave®, and Wavelight Allegro Analyzer®. Results were compared and analyzed in regards to the lower and HOA, to the different wavefront sensing devices and software, Tscherning and Hartmann–Shack and between the Fourier and Zernike algorithms. Statistical analysis included Bland-Altman plots, Intraclass Correlation Coefficient (ICC), multiple comparison tests with Analysis of Variance and Kruskal-Wallis. Significant level was set to *p*<0.05 and alpha level correction was adjusted under the Bonferroni criteria.

**Results:**

Most measurements of all 4 aberrometers were comparable. However, statistically significant differences were found between the aberrometers in total HOA (tHOA), spherical aberration (SA), horizontal coma and astigmatism (2,2). LADARwave and Wavescan showed significant differences in tHOA (P<0.001, ICC = 0.549, LoA = 0.19±0.5) and in SA (P<0.001, ICC = 0.733, LoA = 0.16±0.37). Wavescan showed a significant difference compared to Zywave (p<0.001, ICC = 0.920, LoA = 0.09±0.13) in SA. Comparisons between Allegro Analyzer and Zywave demonstrated significant differences in both Horizontal Coma (3,1) (p<0.001, ICC = −0.207, LoA = −0.15±0.48) and Astigmatism (2,2) (P = 0.003, ICC = −0.965, LoA = 0.2±2.5). Allegro Analyzer also differed from Wavescan in Horizontal Coma (3,1) (P<0.001, ICC = 0.725, LoA = −0.07±0.25).

**Conclusions:**

Although some measurements were comparable predominately in the lower order aberrations, significant differences were found in the tHOA, SA, horizontal coma and astigmatism. Our analysis suggests that sensor design contributes to agreement in lower order aberrations, and Fourier and Zernike expansion might disagree in higher order aberrations. Therefore, comparison between aberrometers was generally possible with some exceptions in higher order measurements.

## Introduction

Last decade technological advancements in aberrometry have revolutionized wavefront-based corneal refractive surgery [1], [2]. Excimer laser technology allowed implementation of wavefront-guided treatments for correction of both lower and higher order aberrations, providing additional benefit in highly aberrated eyes.^3^ Diverse sensor technologies are used in wavefront analysis and different mathematical techniques are employed for wavefront error calculations [4], [5]. Companies designing wavefront aberrometers for the clinical setting implement some of these techniques and methods [3]. Such systems are routinely used as part of the refractive surgery consultation and decision making process [6].

Aberrometers incorporate wavefront analysis to define the refractive parameters of the eye [7]. A wavefront aberration is defined as the deviation of a reflected wave to a reference unaberrated wave [3], [8]. The most common metric in use today is the Root Mean Square (RMS) wavefront error, which is defined as the root square of the wavefront variance over the pupil size of interest [9]. Some visual disturbances such as night vision halos and glare have been associated with highly aberrated eyes [10]. The ability to measure and correct these wavefront abnormalities can provide a benefit in customizing a refractive procedure and in the enhancement of iatrogenically induced aberrations after surgery [11].

There are four main techniques for measuring wavefront aberrations in the eye.[12]–[15] Three of these are based on objective measuring techniques and include: Hartmann-Shack (the most popular), Tscherning and Laser Ray Tracing, while one is based on subjective measurements of the ingoing light, called the spatially resolved [13]. This study utilized two different techniques, Hartmann-Shack and Tscherning. The first measures an outgoing light formed by a laser and reflected by the retina back to a charged-coupled device (CCD) camera using a lenslet configuration [14], while the latter measures an ingoing light formed by a laser grid arrangement reflected by the retina [15].

Regarding mathematical techniques, Zernike and Fourier expansion series polynomials are used in modern optics to describe the optical surface in three dimensions and to quantify these optical abnormalities, referred to as aberrations [16], [17]. Fourier analysis has been available in engineering since the beginning of the 19^th^ century, while Zernike has been described only in the last few decades. Advantages and disadvantages are associated with each technique, mostly related to the processing power and the magnitude of the optical aberration [18].

The purpose of this study was to compare the agreement between 4 different aberrometers using the same reference. All measurements were undertaken in the same eyes, enabling for direct comparisons. The aberrometers used were: the Alcon LADARWave®, the Visx WaveScan®, the B & L Zywave®, and the Wavelight Allegro Analyzer®. Lower and higher order aberrations were compared while controlling the co-effect of the wavefront sensor design, Tscherning (Allegro Analyzer) and Hartmann–Shack (LADARWave, WaveScan, Zywave) and the algorithms for wavefront decomposition, Fourier (WaveScan) and Zernike (LADARWave, Allegro Analyzer, Zywave).

## Methods

We conducted a prospective, cross-sectional study, in a controlled, single-blinded fashion. Forty-two eyes of 21 healthy individuals were included in the study. All subjects were recruited from the Cornea Service of the Massachusetts Eye & Ear Infirmary, Boston, Massachusetts, USA. This study was Health Insurance Portability and Accountability Act (HIPAA)-compliant, adhered to the tenets of the Declaration of Helsinki, and obtained approval of the Institutional Review Board (IRB)/Ethics Committee of the Massachusetts Eye & Ear Infirmary, Boston, Massachusetts, USA. Written informed consent was obtained from all subjects after a detailed explanation of the nature of the study.

All patients underwent a comprehensive eye examination. Detailed review of ophthalmic history, ocular medication, refraction, best-corrected visual acuity, slit-lamp biomicroscopy (with evaluation of the condition of the lid/lashes, conjunctiva, cornea, anterior chamber, iris/pupil, lens, vitreous, macula, optic nerve) and corneal topography was performed. Only adult patients, in generally good and stable health with no ocular abnormality other than refractive error, were included in the study. The patients must not have worn contact lens prior to the study (hard or gas permeable lenses for at least 3 weeks and soft lenses for at least 3 days) and were able to fixate steadily. Patients were excluded when they had history of ocular surgery, trauma and infectious disease, myopia or hyperopia >7.0 D and astigmatism >3.0 D, abnormal corneal topography (e.g. keratoconus), pupil size bellow 6.0 mm under mydriasis, ocular media opacities, ocular movement abnormalities and pregnancy or lactation.

### Aberrometry

Aberrometry was performed in each eye at a single visit with: Alcon LADARWave® (Alcon, Fort Worth, Texas, USA), Visx WaveScan® (VISX, Santa Clara, CA, USA), B & L Zywave® (Baush & Lomb, Rochester, NY, USA), and Wavelight Allegro Analyzer® (Wavelight, Erlangen, Germany). A minimum of 3 readings per eye were obtained with each aberrometer. Patients had a 15 minutes interval between readings with different devices and one drop of lubricant was applied. Subjects were encouraged to blink. Also, head positioning and eye alignment were confirmed before measurements. Natural pupil dilation was achieved under scotopic light condition. All subjects were dilated after Wavescan readings and initial LADARwave exam with Tropicamide 1% for the other three devices, according to the manufacturer’s instructions, starting with Allegro Analyzer (mid-dilated), followed by Zywave and LADARwave.

Lower and higher order aberrations were analyzed and compared between the 4 analyzers. In particular, refraction parameters, defocus (2,0), astigmatism (2,−2 and 2,2) vertical and horizontal coma (3,−1 and 3,1), trefoil (3,−3 and 3,3), spherical aberration (SA) (4,0) and the root mean square (RMS) error of the total aberration and the total higher order aberrations (tHOA) were assessed. Statistical analysis included Bland-Altman plots, Intraclass Correlation Coefficient (ICC), independent comparisons with Mann-Whitney test and multiple comparison tests with Analysis of Variance (ANOVA) and Kruskal-Wallis. Significant level was set to 0.05 and alpha level correction was applied under the Bonferroni criteria for multiple comparisons.

Alcon LADARWave® uses the Hartmann-Shack principle to measure aberrations in the eye. The device software measures the displacement of each focused dot from its ideal location, that is, the pattern generated by a perfectly flat plane wave, and uses this information to calculate the slope of the intact wavefront at each lenslet location. The software then uses this slope data to generate a mathematical description of the original wavefront profile. LADARWave uses a light source of 820 nm. For a 6-mm diameter pupil, it results in 170 wavefront samples at the sensor. This large body of data permits calculation of wavefront aberrations up to the eighth order. The device can measure wavefronts between +15.0 and −15.0 D. The unit can also measure up to 8.0 D of astigmatism. A Zernike expansion series polynomial is used to describe the complex three-dimensional surface [19].

Visx Wavescan® also measures the refractive error and wavefront aberrations of the human eye using a Hartmann-Shack wavefront principle. A small spot of laser light at 785 nm is projected onto the retina and reflects back through the pupil. The WaveScan system can measure spherical refractive errors between −12.0 D and +9.0 D, cylindrical refractive errors up to 5.0 D, and higher-order aberrations up to sixth-order. The WaveScan Fourier wavefront system is capable of reconstructing very complex three-dimensional surfaces with little processing power compared to Zernike expansion. The algorithm provides the ability of peripheral data representation using a multi term polynomial, which means it captures wavefront information in patients with larger pupils with highly aberrated optics and can treat higher and lower aberrations up to 7-mm diameter pupil [19].

B & L Zywave® is another device that measures wavefront aberrations based on the Hartmann-Shack principle. It uses a wavelength of 780 nm and measures approximately 75 locations within the pupil. Up to fifth-order Zernike coefficients are included in the measurements. The Zywave system automatically measures the pupil size at the moment the wavefront image is captured and measures refractive errors over a range of +8.0 D to −14.0 D and up to 5.0 D cylinder. Similar to LADARWave, Zywave implements Zernike expansion for three-dimensional representation of surfaces. [20], [21].

Wavelight Allegro Analyzer® uses a Tscherning sensor architecture in order to capture the ocular wavefront. The Allegro Analyzer projects a grid pattern to the retina. This image is observed using a dot patterned mask and captured by a CCD camera. The distortion of the grid pattern enables calculation of the optical aberrations. A sixth-order Zernike expansion series is utilized using a laser of 660 nm in wavelength. One hundred and seventy wavefront samples are acquired in a dioptric range of +6.0D to −12.0 and up to 6.0D of cylinder [21].

## Results

**Figure 1:**
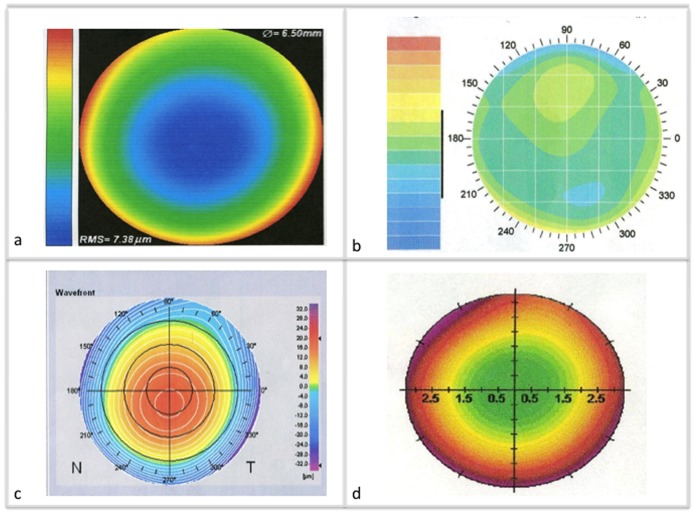
Representation of aberration maps with all four devices. Left eye examination of a patient. LADARWave® (a), Visx WaveScan® (b), Zywave® (c), Allegro Analyzer® (d).

**Figure 2:**
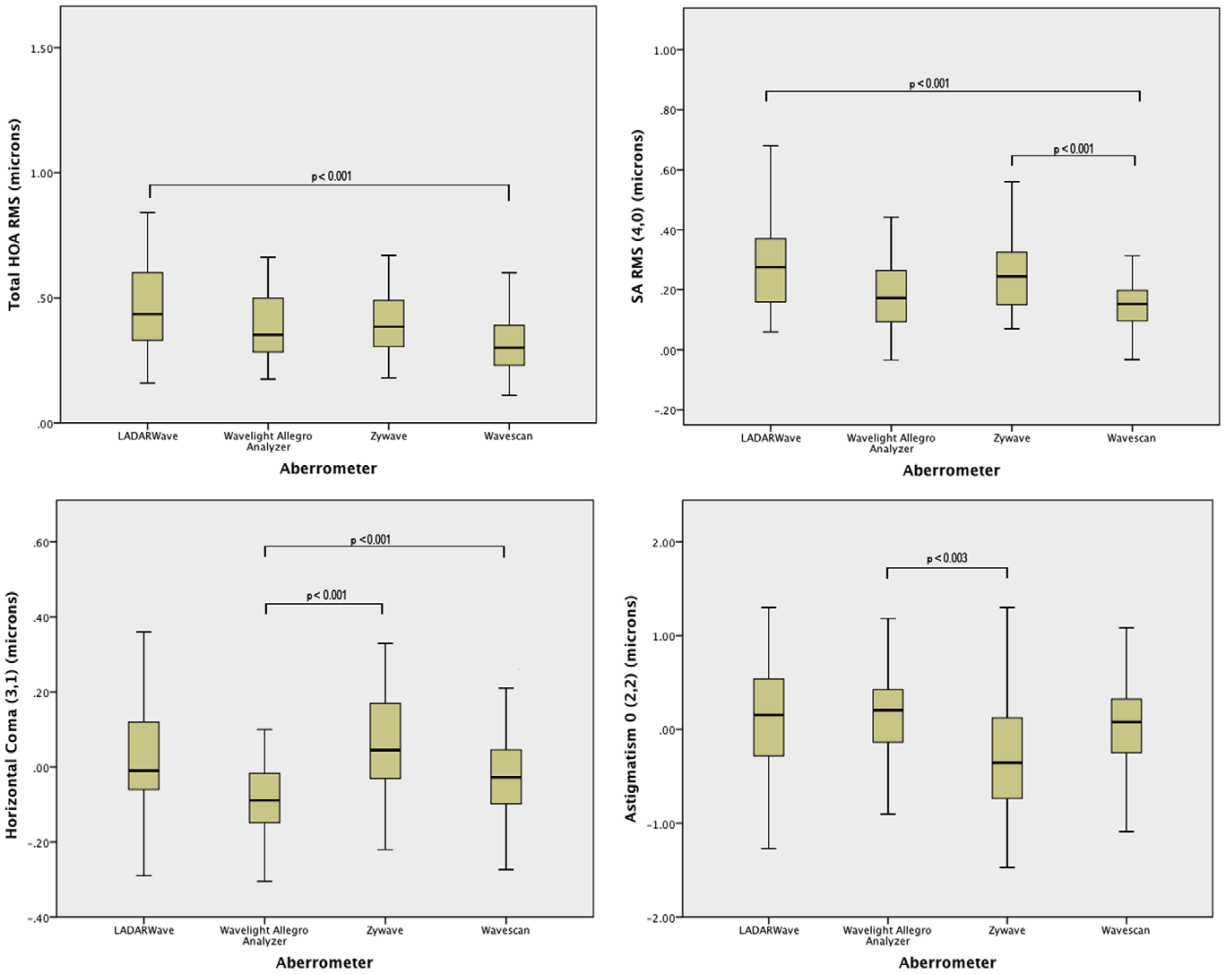
Comparison of aberrations between four aberrometers. Boxplots comparing the distribution of the data. The box contains 50% of the data, the horizontal line inside the box indicates the median value. The whiskers represent the range of the data. Statistically significant difference was found between the 4 aberrometers: A) Total Higher Order Aberrations (tHOA), B) Spherical aberration (SA), C) Horizontal Coma D) Astigmatism (2,2). P-values for multiple comparison tests are represented with connecting lines. RMS = Root mean square.

We compared the measurements of lower and higher order aberrations between the 4 analyzers. Displays of the different aberrometers are illustrated in Figure 1. The summary data of four aberrometers with comparison of wavefront measurements is presented in Table 1. Lower order aberrations between the 4 aberrometers were in agreement with no significant difference in defocus (2,0) (p = 0.189) and oblique astigmatism (2,−2) (p = 0.853), with the exception of astigmatism (2,2) (P = 0.011). Higher order aberrations such as vertical coma (3,−1) (p = 0.209), trefoil (3,−3 and 3,3) (p = 0.170 and p = 0.065) and the root mean square (RMS) error of the total aberration (p = 0.621) did not show a significant difference. However, statistically significant differences were measured between the aberrometers regarding total HOA (p<0.001), SA (4,0) (p<0.001), and horizontal coma (3,1) (p<0.001). Graphic representation of the comparison of aberrations between the four aberrometers is shown in Figure 2 with boxplots.

**Table 1:** Descriptive statistics.

	LADARWave®	Allegro Analyzer®	Zywave®	Wavescan®	P-Value
WF Rx Sph	−1.085±2.055	−1.228±1.939	−0.613±2.094	−0.996±1.873	.316
WF Rx Cyl	−0.595±0.378	−0.539±0.356	−0.673±0.439	−0.560±0.459	.459
Total RMS	3.374±2.952	2.990±2.433	2.576±2.137	2.645±2.009	.621
**tHOA RMS**	**0.503±0.255**	**0.399±0.154**	**0.401±0.132**	**0.332±0.132**	**.001** *
Defocus (2,0)	2.946±3.255	2.286±2.956	1.821±2.699	2.089±2.408	.189
Astigmatism (2, −2)	0.017±0.359	0.060±0.364	−0.007±0.354	0.011±0.333	.853
**Astigmatism (2,2)**	**0.056±0.649**	**0.094±0.587**	−**0.312±0.602**	**0.018±0.583**	**.011** *
Coma (3, −1)	−0.108±0.230	−0.072±0.152	−0.024±0.113	−0.072±126	.209
**Coma (3,1)**	**0.022±0.167**	−**0.084±0.127**	**0.074±0.163**	−**0.010±0.127**	**.001** *
Trefoil (3, −3)	−0.103±0.155	−0.087±0.147	−0.038±0.120	−0.077±0.107	.170
Trefoil (3,3)	0.003±0.160	0.017±0.158	0.075±0.119	0.003±0.107	.065
**SA RMS (4,0)**	**0.293±0.227**	**0.182±0.156**	**0.252±0.126**	**0.157±0.144**	**.001** *

Summary of four aberrometers with comparison of wavefront measurements. WF = wavefront; Rx = Refraction; Sph = Spherical; Cyl = Cylinder; RMS = Root mean square; tHOA = Total Higher Order Aberration; SA = Spherical Aberration.Data is shown as the mean ± SD (in micrometers).

* Significant P<0.05.

**Figure 3:**
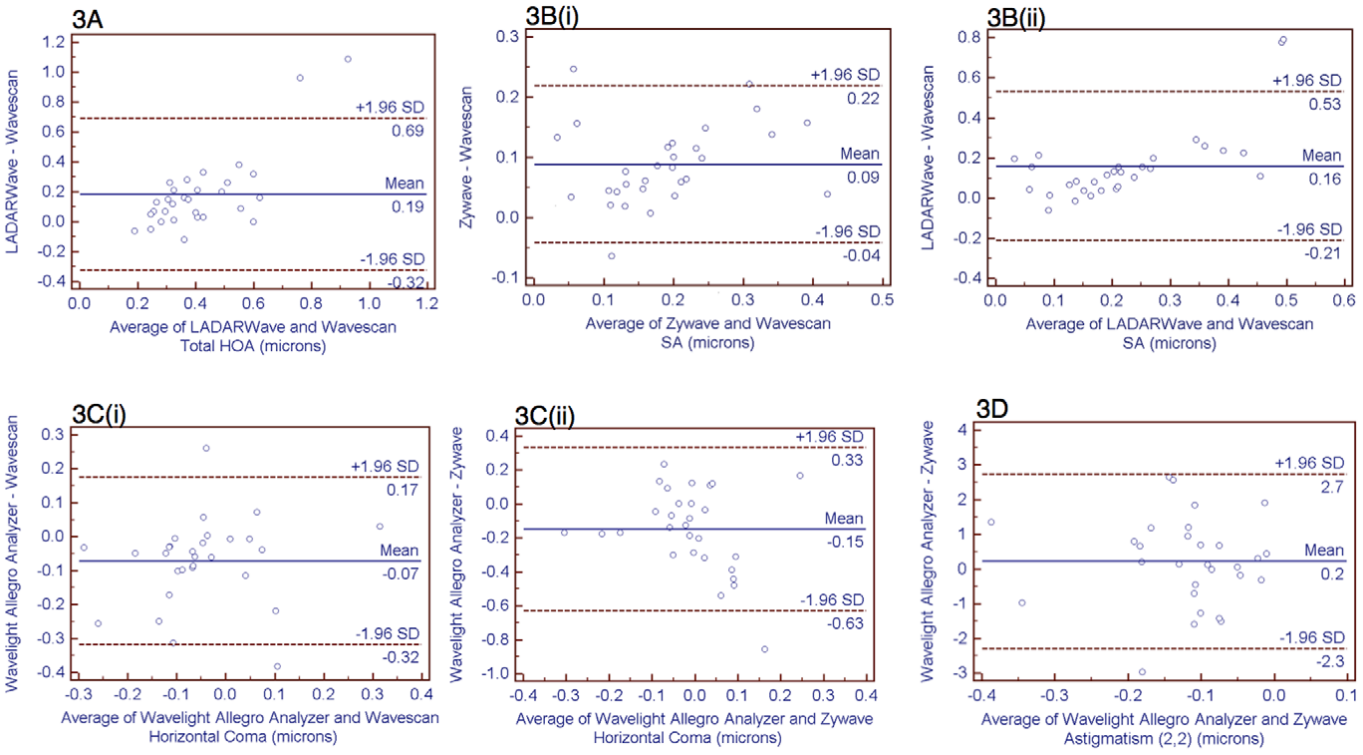
Bland-Altman plots. Diagrams of agreement between the aberrometers. The x-axis represents the mean aberration of two aberrometers, while the y-axis shows the mean difference between the two same aberrometers. The upper and lower horizontal lines represent the upper and the lower limits of agreement (mean difference ±1.96 SD), respectively. The middle line represents the mean difference between the aberrometers. Figure 3A. Correlation between Aberrometers for Total Higher Order Aberrations HOA). Figure 3B(i)(ii). Correlation between Aberrometers for Spherical Aberration (SA). Figure 3C(i)(ii). Correlation between Aberrometers for Horizontal Coma. Figure 3D. Correlation between Aberrometers for Astigmatism (2,2).

Table 2 outlines the significant differences between aberrometer data acquisition and data analysis showing independent comparisons and ICC. Figure 3 shows Bland Altmann difference plots with Limits of agreement (LoA, mean difference ±1.96 SD) of each significantly different independent comparison. LADARwave and Wavescan showed significant difference in tHOA (p<0.001, ICC = 0.549, LoA = 0.19±0.5) (Table 2, Figure 3A) and in SA (p<0.001, ICC = 0.733, LoA = 0.16±0.37) (Table 2, Figure 3B). Although there was a difference between Wavescan and Zywave measurements (p<0.001, LoA = 0.09±0.13), they showed good agreement (ICC = 0.920) in SA (Table 2, Figure 3B).

**Table 2:** Statistical significant differences between aberrometers for total higher order aberrations (tHOA), spherical aberration (SA), horizontal coma and astigmatism (2,2).

	P-Value	ICC	CI 95%
**Total HOA**			
LADARWave (HS,Z) vs Wavescan (HS,F)	.001	.549	0.161 to 0.757
**SA**			
LADARWave (HS,Z) vs Wavescan (HS,F)	.001	.733	0.504 to 0.857
Zywave (HS,Z) vs Wavescan (HS,F)	.001	.920	0.849 to 0.958
**Horizontal Coma**			
Allegro Analyzer (T,Z) vs Zywave (HS,Z)	.001	−.207	−1.302 to 0.367
Allegro Analyzer (T,Z) vs Wavescan (HS,F)	.001	.725	0.485 to 0.853
**Astigmatism (2,2)**			
Allegro Analyzer (T,Z) vs Zywave (HS,Z)	.003	−.965	−9.834 to −0.927

Significant P-Value <0.01 by Mann-Whitney (Bonferroni adjusted). ICC = Intraclass correlation coefficient. CI = Confidence interval. HS = Hartmann-Shack, T = Tscherning, Z = Zernike, F = Fourier.

Comparisons between Allegro Analyzer and Zywave demonstrated significant differences in both horizontal coma (3,3) (p<0.001, ICC = −0.207, LoA = −0.15±0.48) (Table 2, Figure 3C) and astigmatism (2,2) (p = 0.003, ICC = −0.965, LoA = 0.2±2.5) (Table 2, Figure 3D). Allegro Analyzer also differed from the Wavescan in horizontal coma (3,3) (p<0.001, ICC = 0.725, LoA = −0.07±0.25) (Table 2, Figure 3C).

## Discussion

There are few studies comparing aberrometers in the literature, and most of those have emphasized the differences amongst them. In addition, direct comparison between aberrometers is not possible prior to establishing a common measuring reference. In the current study, we used the same sample to compared 4 different aberrometers according to manufacturer’s recommendations. This is the first study to simultaneously assess the agreement between aberrometers while assessing for the effect of the aberrometers design.

Aberrations were evaluated according to the two types of wavefront sensors: Hartman-Shack (LADARWave, WaveScan, Zywave) and the Tscherning based wavefront analyzers (Allegro Analyzer). Furthermore, evaluation was undertaken between Fourier (WaveScan) and Zernike (LADARWave, Allegro Analyzer, Zywave) expansion series polynomials, which are used to describe the optical geometry of the visual system in three dimensions and are represented as lower and higher order aberrations. Due to the lack of a gold standard method to measure ocular aberrations, this study did not focus on determining which device exhibited the most objective measurements.

In general, there was good agreement between both lower and higher order aberrations for all 4 wavefront analyzers in this study, summarized in Table 1. The 4 aberrometers showed similarities in the lower order aberrations including defocus (2,0) (p = 0.189), and oblique astigmatism (2,−2) (p = 0.853). Also, higher order aberrations such as vertical coma (3,−1) (p = 0.209), trefoil (3,−3 and 3,3) (p = 0.170 and p = 0.065) and the root mean square (RMS) error of the total aberration (p = 0.621) showed to be comparable. However, significant differences were found between the aberrometers in regards to tHOA, SA, horizontal coma and astigmatism (2,2) (Table 2). These differences were then analyzed and graphically represented as Bland Altmann plots (Figure 3). Such graphics provide the mean difference and the standard deviation of the differences between devices. Different from correlation coefficients, where any comparison can be highly correlated even when there is a consistent bias in measurements, our analysis showed a wide spread sample distribution of independent comparisons for each one of the aberrations that were statistically different.

The overall analysis showed that the Allegro Analyzer measurements were different compared to Zywave and Wavescan for lower order astigmatism aberration and horizontal coma. It is important to note that Allegro Analyzer uses Tscherning sensor architecture while the other two aberrometers employ Hartmann-Shack, suggesting that the sensor design particularly contributes to agreement in lower order aberrations.

Some studies have tried to compare measurement acquisition with different devices focusing their analysis on either repeatability or interchangeability. Most of these studies have shown good correlation between aberrometers, although some parameters had poor agreement [21]–[24]. Visser et al., showed that Hartmann-Shack aberrometers had the best repeatability in regards to total ocular aberrations comparing measurements between Irx3 (Hartmann-Shack), Keratron (Hartmann-Shack), iTrace (Tshcerning), and OPD-Scan (Automated Retinoscopy) analyzers. However, in direct comparison of measurements, the ocular aberrations obtained with the four analyzers showed significant differences in astigmatism (2,2), defocus (2,0), trefoil (3,−3 and 3,3), and spherical aberration (4,0) [23]. In another study by Rozema et al., with six different aberrometers, similar results were obtained [21], [24].

One explanation for this discrepancy might be the differences in the algorithm to locate either the chief ray of each lenslet image or the pupil center. Consequently, any disparity of mathematical calculation, used by each device, can give slightly different results [9].

The comparison between Fourier and Zernike was done in order to elucidate possible differences in the mathematical reconstruction of the three-dimensional surface. The Wavescan was the only device using Fourier expansion among the four aberrometers in our study. Interestingly, Wavescan showed statistically significant differences compared to LADARwave and Zywave, in tHOA and SA (p<0.001), raising the idea that mathematical analysis may contribute to differences in higher order aberration measurements among aberrometers. Although some authors have been advocating that these differences are more important in engineering rather than in clinical setting, this observation allows us to suggest that Fourier and Zernike analysis might differ in regards to higher order aberrations.

As shown by Klyce et al., Zernike appears to clinically underestimate the amount of higher order aberrations in highly aberrated eyes, such as Keratoconus [25]. Fourier polynomials were suggested as an alternative to decompose wavefront maps and its expansion is felt to be more reliable and efficient in representing the overall wavefront map. Liang et.al have also found differences between Wavescan (Fourier)-Zywave (Zernike), and between Wavescan (Fourier)-LADARWave (Zernike) in tHOA and SA [26]. Similar findings were shown in subsequent studies where data comparison between LADARWave (Zernike) and Wavescan (Fourier) have also demonstrated differences in terms of tHOA and SA [19], [27].

Advantages of Fourier analysis can be attributed to the simplicity of mathematical calculations, but clinically, minimum differences can be seen between the two methods. Zernike expansion conveniently represents tilt, prism, sphere, cylinder, spherical aberration and coma, traditionally used in ophthalmology. Fourier transform does not have such single term representation (Zernike polynomials) and requires the sum of multiple Fourier terms to represent aberrations. However, Fourier has the capability to calculate complex and highly irregular surfaces with more precision than Zernike, while Zernike exhibits difficulties in mapping surface irregularities in the periphery of the analyzed area. Additionally, Zernike requires a higher amount of computing power relative to Fourier [4], [18].

Another parameter that can interfere with results is the need for pupillary dilation for wavefront aberrometry by some analyzers. The location of the pupil center and the pupil size are essential factors in wavefront analysis [28], [29]. Pupil importance relies in the control of light intensity, which defines the point spread function of the visual system.^30^ Therefore, either pharmacologic pupil dilation or pupil displacement can contribute to the increase of higher order aberrations, with third-order coma and higher order spherical aberration induction [29], [30]. In addition, increase of aberration in the optical system can be attributed to changes of lens’ accommodation [31]. Beside the expected differences that theoretically should be found between a dilated pupil compared to a mesopic pupil acquired aberrometry, we decided to follow the manufacturer’s recommendation in order to obtain “real life” results similar to the clinical setting, and compare them to how a refractive surgeon would evaluate them.

In summary, the purpose of this study was to evaluate which variables were comparable and which were different between 4 aberrometers using the same eyes. It has been previously documented that data comparison across aberrometers is not straightforward and requires careful analysis, even when assessing related devices. Our analysis showed similarities between all aberrometers, particularly measuring lower order aberrations. However, significant differences found in total HOA, SA, horizontal coma and astigmatism suggest that certain measurements are not always consistent between devices, and hence, should be interpreted carefully. We believe that these differences can be attributed to design variations between the aberrometers, such as sensor architecture and the wavefront decomposition algorithm.

### What was Known

- There are few studies comparing aberrometers in the literature, most of them have tried to compare measurement acquisition with different devices focusing their analysis on either repeatability or interchangeability.
- Poor agreement between different devices has been shown to occur when comparing higher order aberrations

### What this Paper Adds

- This is the first study to simultaneously assess the agreement between these 4 specific aberrometers while also assessing for the effect of the aberrometers design.
- In general, there was good agreement between both lower and higher order aberrations for all 4 wavefront analyzers in this study.
- However differences were found, and our analysis suggests that sensor design (Hartmann-Shack and Tscherning) contributes to agreement in lower order aberrations, and Fourier and Zernike expansion might disagree in higher order aberrations.
